# Effect of Connective Tissue Graft as an Adjunct to Guided Bone Regeneration in the Surgical Treatment of Peri‐Implantitis: A Dual‐Center Randomized Controlled Trial

**DOI:** 10.1111/clr.70093

**Published:** 2026-01-31

**Authors:** Lucrezia Paterno Holtzman, Iva Milinkovic, Marija Vuckovic, Chiara Malpassi, Marla Cuppini, Alex Solderer, Zoran Aleksic, Luca Cordaro

**Affiliations:** ^1^ Periodontology and Prosthodontics, George Eastman Dental Hospital University Policlinic “La Sapienza” Rome Italy; ^2^ Dipartimento di Scienze Orali e Maxillofaciali Università “La Sapienza” Rome Italy; ^3^ Implant Center, School of Dental Medicine University of Belgrade Belgrade Republic of Serbia; ^4^ Department of Periodontology and Oral Medicine, School of Dental Medicine University of Belgrade Belgrade Serbia; ^5^ Department of Medical Biotechnologies, Unit of Periodontology, Endodontology and Restorative Dentistry University of Siena Siena Italy; ^6^ Clinic of Conservative and Preventive Dentistry, Center for Dental Medicine University of Zurich Zurich Switzerland

**Keywords:** connective tissue graft, dental implants, guided bone regeneration, peri‐implantitis

## Abstract

**Objectives:**

To evaluate whether adding a connective tissue graft (CTG) to guided bone regeneration (GBR) improves clinical and radiographic outcomes in surgical peri‐implantitis treatment.

**Materials and Methods:**

Thirty‐two patients with peri‐implantitis were randomly assigned to receive GBR and CTG (test group, TG) or GBR alone (control group, CG). Clinical and radiographic parameters were recorded at baseline, 6, and 12 months. The primary outcome was the change in clinical attachment level (CAL), while secondary outcomes included pocket probing depth (PPD), bleeding on probing (BoP), plaque index (PI), keratinized mucosa width (KMW), mucosal thickness (MT), recession (REC), suppuration (SUP), marginal bone levels (MBL), bone defect morphology, and disease resolution (DR).

**Results:**

At 12 months, CAL gain was significantly higher in TG compared with CG (3.21 ± 1.57 mm vs. 1.65 ± 1.28 mm; *p* = 0.022), and TG achieved significantly greater increase in KMW (2.25 ± 2.89 mm vs. 0.26 ± 1.49 mm; *p* = 0.010). Both groups showed significant PPD reduction, with a greater, though not statistically significant, improvement comparing TG with CG (3.25 ± 1.59 mm vs. 1.97 ± 1.23 mm; *p* = 0.052). Additionally, MBL improved significantly in both groups (*p* < 0.001), with higher gains in TG (*p* < 0.001). However, DR was comparable between the two groups.

**Conclusions:**

GBR effectively improves peri‐implant parameters after 1 year. Adding a CTG enhances CAL and KMW gains and may promote more favorable bone levels, although the impact on DR remains inconclusive. Long‐term studies are warranted to confirm these findings.

**Trial Registration:**

ClinicalTrials.gov NCT04323540

## 1 Introduction

The role of keratinized mucosa (KM) on implant health has long been discussed. Evidence suggests that a KM width less than 2 mm is associated with severe forms of peri‐implant disease and that, in its absence, implants tend to exhibit worse clinical parameters (Ravidà et al. 2020; Roccuzzo et al. 2016). A recent 10‐year follow‐up study has additionally confirmed that the absence of buccal KM is associated with bleeding on probing around implants (Mancini et al. 2024). Various surgical treatments have been explored for peri‐implantitis. Among these, reconstructive therapy appears to be more predictable compared to nonsurgical modalities (Donos et al. 2023; Solderer et al. 2024). However, reconstructive approaches have often been investigated in areas with adequate KM (≥ 2 mm), e.g., (Regidor et al. 2023; Monje et al. 2025; Galarraga‐Vinueza et al. 2020; Soldini et al. 2025). In addition, the surgical handling characteristics of KM are often better compared to lining mucosa (Paternò Holtzman et al. 2024; Khoury et al. 2019), which is frequently found around implants with peri‐implantitis (Gharpure et al. 2021).

In the GBR technique, the importance of wound stability is particularly crucial for the success of the procedure (Wang and Boyapati 2006). Adding a subepithelial connective tissue graft (sCTG), the current gold standard in soft tissue grafting, beneath the flap may provide a clinical benefit due to improved wound margin stability and, consequently, improve the outcome of reconstructive procedures (Thoma et al. 2009; Tavelli et al. 2021). Additionally, a case series combining sCTG with a reconstructive technique for peri‐implantitis treatment reported reduced postsurgical recession and improved stability of the buccal soft tissue margin, contributing to better esthetic outcomes (Schwarz et al. 2014). Furthermore, the addition of a sCTG has been proven beneficial in improving the tensile strength of the wound margins and overall flap stability (Burkhardt R, Ruiz Magaz V, Hämmerle CH, Lang NP. 2016). To the best of our knowledge, an RCT comparing reconstructive peri‐implantitis treatment with or without sCTG has not been published yet.

Taking the aforementioned into consideration, this study aimed to evaluate whether adding an autologous subepithelial connective tissue graft (sCTG) combined with a reconstructive peri‐implantitis modality (GBR) could achieve better outcomes in terms of CAL at 12 months (primary outcome), as well as improvements in other surrogate markers of peri‐implant health, compared to GBR alone.

## 2 Materials and Methods

The present study was designed as a multicenter, parallel‐group, randomized, controlled trial with a 1:1 allocation ratio. The study was conducted in compliance with the investigation plan, the current version of the Declaration of Helsinki, ISO EN 14155, as well as national legal and regulatory requirements. The protocol received approval from the responsible authorities (Approvazione 6109 Comitato Etico Università “La Sapienza” and 36/9 Ethic Committee of the School of Dental Medicine, University of Belgrade, Approval from April 20, 2020) and was registered in the Clinicaltrials.gov database on March 23, 2020 (no. NCT04323540). CONSORT guidelines were adhered to as per protocol (Hopewell et al. 2025). Additionally, the study is in accordance with the recommendations contained in the VIII EFP Consensus Report on Clinical Research on Peri‐implant Diseases (Sanz and Chapple 2012). The case definitions used are consistent with those outlined in the 2017 World Workshop (Renvert et al. 2018).

### 2.1 Study Setting

The study was conducted in the Periodontology and Prosthodontics Department of the Eastman Dental Hospital, Rome (Italy) and in the Department of Periodontology and Oral Medicine, School of Dental Medicine, University of Belgrade (Serbia).

### 2.2 Study Population

**FIGURE 1:**
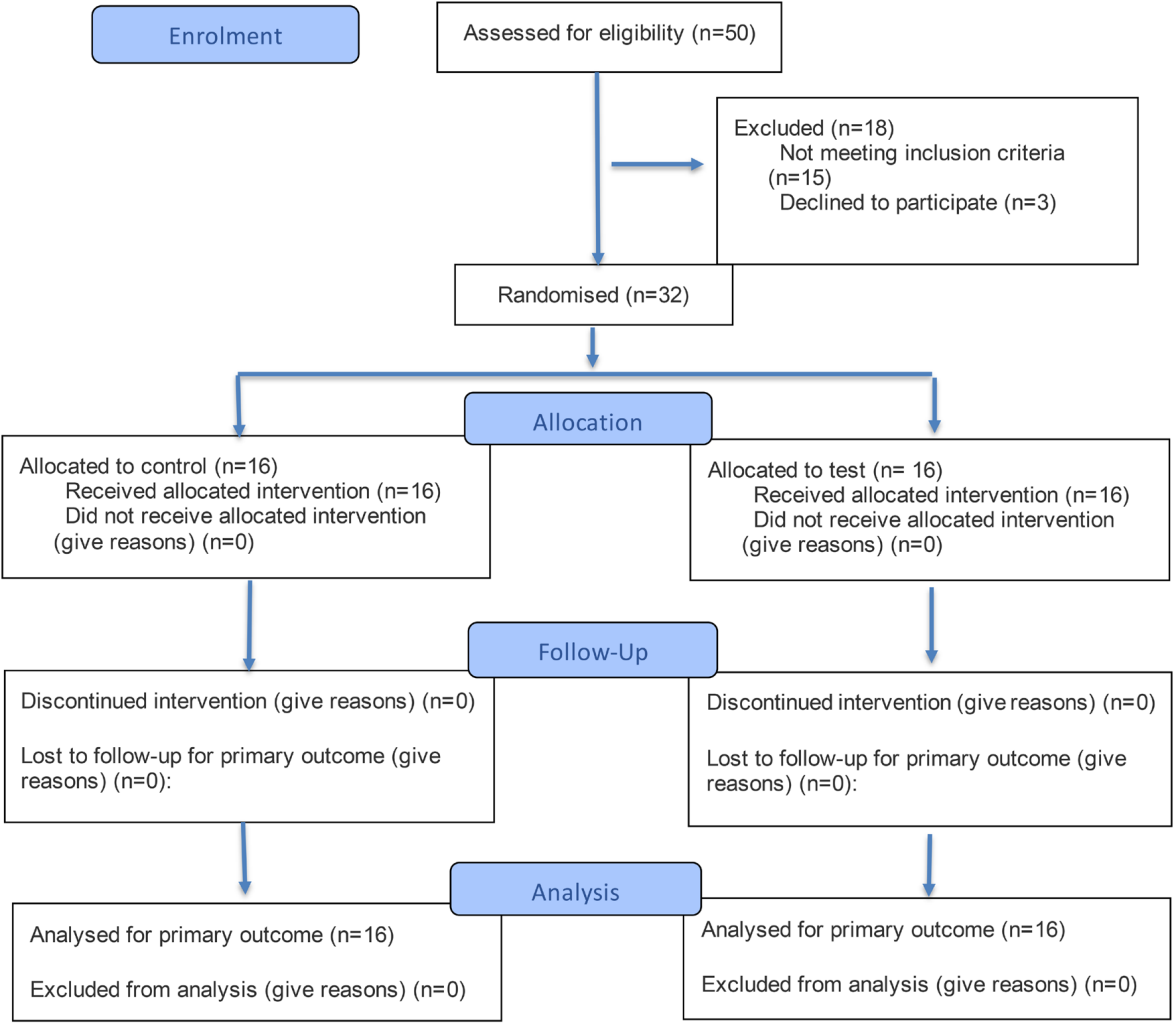
CONSORT 2025 flow diagram. Citation: Hopewell S, Chan AW, Collins GS, Hróbjartsson A, Moher D, Schulz KF, et al. CONSORT 2025 Statement: Updated guideline for reporting randomised trials. BMJ. 2025; 388: E081123. https://doi.org/10.1136/bmj‐2024‐081123 2025 Hopewell et al. This is an Open Access article distributed under the terms of the Creative Commons Attribution License (https://creativecommons.org/licenses/by/4.0/), which permits unrestricted use, distribution, and reproduction in any medium, provided the original work is properly cited.

Thirty‐two patients were included, with sixteen patients assigned to each treatment group (Figure 1). All patients met the inclusion and exclusion criteria, prior to inclusion all patients provided their written informed consent.

#### 2.2.1 Inclusion Criteria

- Be able and willing to provide and sign the informed consent form and to comply with study procedures and follow‐up appointments required by the protocol.
- Age ≥ 18 years
- Presence of at least one implant affected with peri‐implantitis, defined as the following condition: at least one site presenting PPD ≥ 6 mm and simultaneous presence of profuse BoP/SUP, and a radiographically documented change in bone loss greater than initial bone remodeling (0.5 mm within the first year of loading). If a baseline radiographic image was absent, the bone level was estimated based on its expected position at the time of implant insertion, and a difference of ≥ 3 mm from that level was considered a sign of peri‐implantitis
- Implants in function (i.e., loaded) for at least 1 year.
- Fixed (screw‐ and cement‐retained) and removable implant‐supported suprastructures.
- Based on the baseline width of the buccal KM, patients would lie in one of two strata, defined as follows:
  i. Strata 1: KM < 2 mm.
  ii. Strata 2: KM ≥ 2 mm.

#### 2.2.2 Exclusion Criteria

Systemic conditions:

- Compromised systemic health, which contraindicated the study procedures.
- Pregnant or nursing women.
- Cigarette smoking > 20 per day
- Systemic conditions compromising healing (i.e., uncontrolled diabetes mellitus). Patients with diabetes mellitus type I or II were asked to provide information regarding their most recent HbA1c values. Only patients with HbA1c < 7% were eligible.
- Patients taking medications known to interfere with gingival or bone metabolism or with collagenopathies/connective tissue disorders.

Local conditions:

- Implants with ≥ 60% of bone loss on at least one aspect of the implant, or clinical mobility.
- Implants already treated previously (with surgical or nonsurgical therapy) for peri‐implantitis.
- Peri‐implant defects with a purely supraosseous component not amenable to reconstructive treatment (determined upon flap reflection)
- Periodontal inflammation (FMBS> 25%).
- Patients with inadequate oral hygiene (FMPS> 20%).

### 2.3 Surgical Interventions, Pre‐ and Postoperative Care

Detailed, personalized oral hygiene instructions were provided, and smoking cessation was encouraged. Suprastructures were examined, and if found to be inadequate or obstructive to proper hygiene access, were corrected when possible. If patients had FMPS ≥ 20% they had to improve oral homecare before being admitted to the study.

All participants underwent an initial phase of nonsurgical treatment 6 weeks prior to surgery consisting in submucosal instrumentation with titanium curettes at the study implant sites (Hu‐Friedy, Mfg. Co LLC., Titanium implant Scalers) and a combination of stainless‐steel curettes (Hu‐Friedy, Mfg. Co LLC.) and ultrasonic devices (Guilin Woodpecker Medical Instrument CO. Ltd. U600) around natural teeth. During the baseline (T_0_) appointment, clinical parameters were recorded, followed by the surgical intervention.

Prior to surgery, all efforts were made to safely remove prosthetic restorations without causing damage. If the reconstruction could not be removed, the surgical intervention was performed with the reconstruction in place. Upon flap reflection, the envelope containing randomization information was opened, and the patient was thus assigned to the test or control group.

#### 2.3.1 Control Group

Subjects assigned to the control group were treated under local anesthesia with intrasulcular buccal and lingual incisions and vertical releasing incisions if the surgeon felt that extending the flap was necessary. Full‐thickness flap reflection followed by complete degranulation of the defect was performed using titanium curettes (Hu‐Friedy, Mfg. Co LLC., Titanium implant Scalers) (Schwarz et al. 2014). The implant surface was thoroughly decontaminated using a titanium brush (de Tapia et al. 2019, 2025; González et al. 2021). This was followed by mechanical cleaning with an air‐polishing device (Air‐flow handy 3.0 Perio, EMS Dental, Nyon, Switzerland) using erythritol powder containing 0.3% (Hentenaar et al. 2022; Bi et al. 2023). Finally, the site was irrigated with sterile saline. The infrabony component of the defect was grafted using a collagenated deproteinized bovine bone mineral block (DBBM‐C) (Bio‐Oss Collagen, Geistlich, Wolhusen, Switzerland), and a resorbable membrane (Biogide, Geistlich, Wolhusen, Switzerland) was trimmed and adapted to cover the defect. The flaps were coronally positioned and sutured.

#### 2.3.2 Test Group

The test group was treated using the same approach and materials as the control group. After membrane positioning, an autogenous epithelial connective tissue graft from the palate was harvested according to the defect dimensions with a thickness of approximately 1.5 mm. The CTG placement was extra‐orally de‐epithelialized, sutured 1 mm apical to the flap margin on the buccal aspect, and fixed to the buccal flap with two horizontal mattress sutures, following the protocol described by Stefanini et al. (2016) (Stefanini et al. 2016). The mesio‐distal dimensions of the graft exceeded the defect by 2 mm on each side. The flap was adapted to the prosthetic abutment/transmucosal component and sutured for complete coverage of the graft. No attempt was made to graft the occlusal aspect using a “poncho” configuration. In both groups, healing was undisturbed by prosthetic reconstruction positioning until 2 weeks postoperatively (only in cases where prostheses were removed).

#### 2.3.3 Perioperative Care

Three days prior to the surgical procedure, patients began antibiotic therapy (1 g of amoxicillin plus clavulanic acid twice daily or—in case of penicillin allergy—clindamycin 150 mg four times daily) and continued this antibiotic regimen for a total of 10 days (Carcuac et al. 2016; Derks et al. 2022). After surgery, patients were instructed to rinse with 0.2% chlorhexidine (Corsodyl GlaxoSmithKline) for 1 min, twice a day for 2 weeks. Sutures were removed 14 days after surgical procedures, and brushing was resumed with a soft‐bristle toothbrush. Full‐mouth supragingival professional hygiene and customized oral hygiene procedures were carried out 1 month after surgery, followed by maintenance sessions every 3 months.

### 2.4 Clinical and Radiographic Measurements

All clinical parameters were recorded at six sites per implant: mesio‐buccal, mid‐buccal, disto‐buccal, mesio‐lingual, mid‐lingual, and disto‐lingual, using a periodontal probe (UNC‐15 Hu‐Friedy, Chicago, IL, USA). Clinical measurements were recorded at baseline (T_0_), 6 months (T_1_), and 12 months (T_2_), and values were represented as the mean of six sites per implant. Radiographic measurements were performed at T_0_ and T_2_ using Rinn XCP kits (Dentsply Italia SRL).

#### 2.4.1 Primary Outcome

Clinical attachment level (CAL): measured as the distance between the prosthetic margin and the bottom of the pocket (mm) at T_0_, T_1_, and T_2_.

#### 2.4.2 Secondary Outcomes

Probing pocket depth (PPD): measured from the mucosal margin to the bottom of the probable pocket (mm), it was assessed at T_0_ and at T_1_, T_2_.

Keratinized mucosa width (KMW): distance measured from mucosal margin to mucogingival junction (mm) at T_0_, T_1_, and T_2_.

Buccal mucosal recession (REC): recorded at T_0_, T_1_, and T_2_ in mm as the linear distance between the restorative margin to the mucosal margin on the buccal aspect. In the case of removable prostheses (for instance, overdentures), the distance was measured from the occlusal surface of the implant/locator or implant/abutment junction to the mucosal margin. If the mucosal margin lay coronal to the reference point, the number was preceded by a negative sign.

Mucosal thickness (MT): measured using the transgingival probing technique, whereby a needle with a silicone stopper was inserted perpendicularly (2 mm apical of the mucosal margin) through the gingiva under local anesthesia until contact with the underlying bone/implant surface was achieved, and the distance from the gingival surface to the bone was recorded (mm).

Bleeding on probing (BoP): binary variable evaluated as presence/absence of bleeding upon peri‐implant probing at T_0_, T_1_, and T_2_.

Suppuration (SUP): binary variable evaluated as presence/absence of suppuration upon peri‐implant probing at T_0_, T_1_, and T_2_.

O'Leary Plaque index (PI): binary variable evaluated as presence/absence of marginal plaque, recorded using a disclosing dye at T_0_, T_1_, and T_2_.

Radiographic Marginal Bone Level (BL): distance between a reference point (implant platform for bone level and smooth‐rough interface for tissue level implants) and the mesial/distal aspects of the implant (mm). The radiographs were taken at T_0_ and T_2_. Standardized radiographs of the implant sites were at visits T_0_ and T_2_ and the films were calibrated based on the known implant diameter and marginal bone level. The distance from the implant shoulder to the bone crest (DIB) was measured at the mesial and distal aspects of the implants with an accuracy of 0.01 mm. Changes in marginal bone levels were evaluated between the different time points.

Peri‐implant bone defects morphology: The morphology of peri‐implant bony defects was intraoperatively classified according to Monje et al. (2019). Only class I or III defects were included. Each class was further subclassified as: (a) dehiscence, (b) 2/3‐wall, or (c) circumferential defects.

Disease resolution: The resolution outcomes defined by the EFP criteria and by the criteria of Derks et al. (2022) were evaluated at T_0_ It was defined as either:

Simultaneous presence of: (i) ≤ 1 BoP, (ii) absence of SUP, (iii) PPD ≤ 5 mm, iv. absence of progressive bone loss compared to pre‐treatment levels (Herrera et al. 2023) (composite definition 1) or,
(i) Implant not lost; (ii) Absence of BoP/SOP on all sites; (iii) PPD < 5 mm on all aspects; (iv) ≤ 1 mm buccal recession of the mucosal margin (composite definition 2) (Derks et al. 2022). Both measures of disease resolution were assessed at T_2_

### 2.5 Additional Parameters Monitored

#### 2.5.1 Early Would Healing Index (EHI)

A score from 1 to 5 was assigned (Wachtel et al. 2003) according to EHI at 1‐ and 2‐weeks, and was assessed by the surgeon (unblinded).

#### 2.5.2 Intraoperative Time (Min)

The time from anesthesia to final suture, not considering the time necessary for prosthesis removal and re‐cementation/delivery (if applicable).

### 2.6 Sample Size Calculation

The sample size calculation was based on the findings of a comparable study by Schwarz et al. (2010). In that study, baseline clinical attachment level (CAL) values were comparable across defect configurations, and mean CAL values at 12 months ranged from approximately 5.1 to 6.4 mm, depending on defect class (Ib, Ic, and Ie). For the purpose of sample size estimation, an overall mean CAL value of 5.8 ± 0.7 mm was calculated by averaging the CAL values reported for the different defect classes. It was assumed that baseline CAL values would be similar between groups as a result of randomization and that a 15% difference in final CAL between groups (e.g., 5.8 mm vs. 6.67 mm) would be clinically significant. A two‐tailed independent samples *t*‐test indicated that 12 patients per group would provide 80% power at a 95% confidence level. To account for an anticipated dropout rate of 30%, 16 patients were planned per group.

Although Schwarz et al. (2010) reported a normal distribution, subsequent analysis of our dataset revealed a non‐normal distribution. Therefore, a nonparametric approach was also considered. The sample size was recalculated for the Mann–Whitney test using the minimum asymptotic relative efficiency (Pitman) to account for the worst‐case efficiency of the Mann–Whitney test relative to the *t*‐test, resulting in a similar requirement of 13 patients per group.

### 2.7 Randomization and Allocation Concealment

In order to ensure equal distribution among KM width values, a potential confounder, a stratified randomization technique was employed. A randomization sequence was prepared by hospital personnel not involved in the study using a computerized random number generator (Random.org; www.random.org). The sequence was concealed by keeping it on a password‐protected computer accessible only to an independent coordinator who was not involved in participant enrollment or treatment. After obtaining informed consent and completing baseline assessments, the enrolling investigator contacted the independent coordinator of each center, who then revealed the treatment assignment to the surgeon, only once the flap had already been reflected. This process ensured that allocation remained concealed until the point of assignment.

### 2.8 Study Personnel

In each clinical center, all surgical procedures were performed by one surgeon (LPH, IM), a blinded outcome assessor made all clinical measurements at baseline and follow‐up visits (CM, MV), and an independent and blinded examiner who performed radiographic measurements (the same for both centers, AS).

### 2.9 Calibration

A probing calibration exercise for the primary outcome, clinical attachment level (CAL), was conducted prior to study initiation. Each examiner underwent standardized training and calibration under supervision, using controlled probing force and uniform measurement protocols to ensure methodological consistency. Furthermore, surgeons had multiple calibration meetings with case discussions to ensure a comparable surgical approach and graft positioning. Intraexaminer reliability was assessed by repeating CAL measurements on the same sample subjects (not included in the study) after 1 week, resulting in an intraexaminer correlation coefficient of 0.98 and an interexaminer correlation coefficient of 0.89.

### 2.10 Statistical Analysis

Descriptive statistics were performed for both categorical variables (absolute and relative frequencies) and continuous variables (mean, standard deviation, median, interquartile range, and range). Due to the non‐normal distribution of most continuous data, nonparametric tests were applied. Group comparisons for continuous and ordinal variables were conducted using the Mann–Whitney *U* test, while associations between categorical variables were examined with the Chi‐square or Fisher's exact test, depending on sample size. To assess longitudinal changes, especially in clinical and radiographic outcomes, the Brunner–Langer nonparametric model was used. Within‐group changes were analyzed using Wilcoxon signed‐rank tests with Bonferroni correction, while between‐group differences were compared at each time point. Binary logistic regression was employed to evaluate the associations between the treatment group or covariates and disease resolution outcomes, yielding odds ratios (ORs) with 95% confidence intervals. Interaction terms were introduced to assess whether the differences between groups varied across KM strata. A significance level of 5% (*α* = 0.05) was used for all analyses. For exploratory purposes, trends with *p*‐values between 0.05 and 0.1 were also reported and interpreted with caution, as they may indicate potential associations that warrant further investigation in studies with larger sample sizes.

## 3 Results

### 3.1 Study Population Characteristics and Implant‐Specific Variables

**FIGURE 2:**
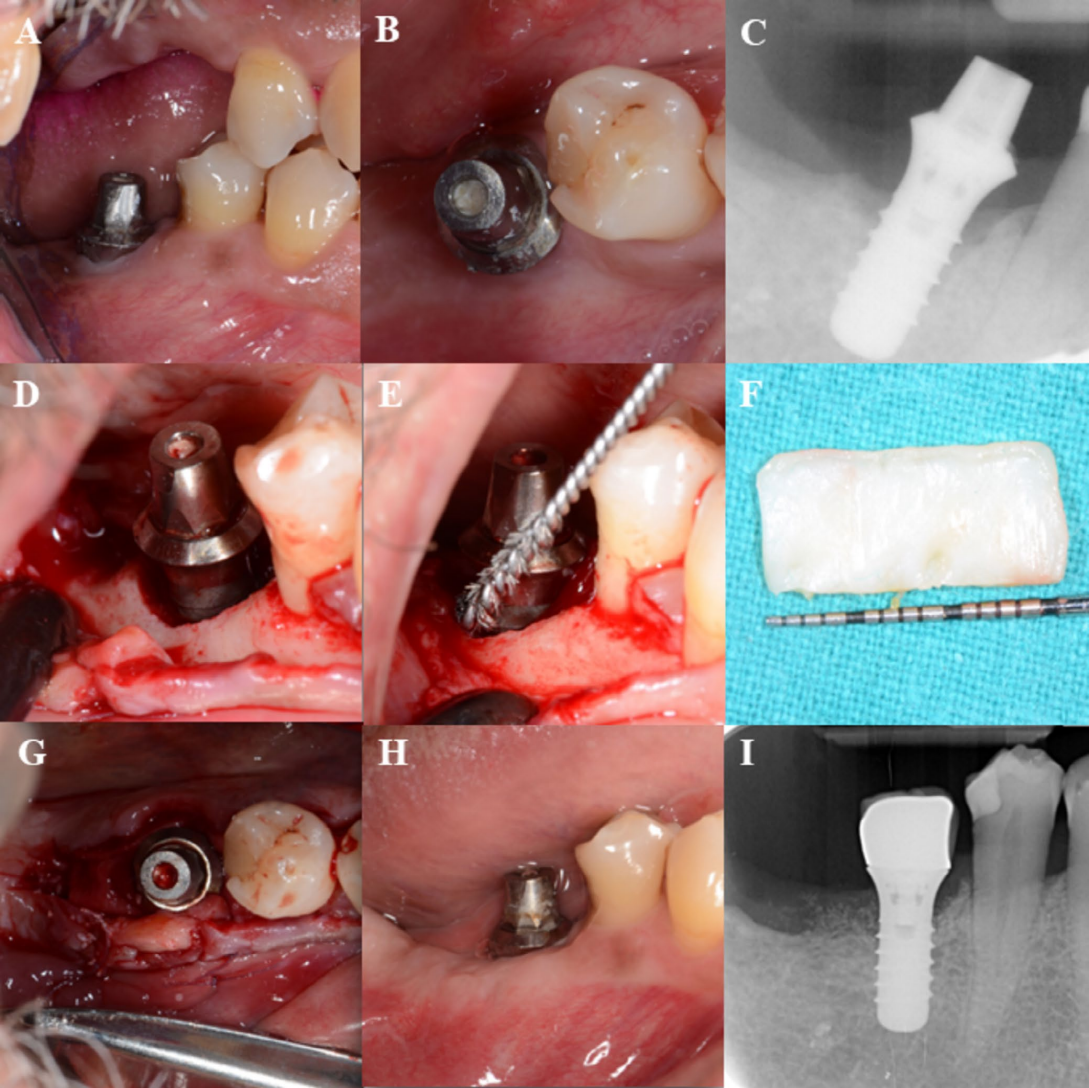
Test group, strata 1—case report, regio 46. (A) Baseline, buccal view; (B) baseline, occlusal view; (C) baseline X‐ray; (D) surgery, flap elevation, buccal view of the peri‐implant bone defect; (E) implant surface decontamination with a Ti brush; (F) De‐epithelialized free‐gingival graft; (G) occlusal view of the implant with collagenated DBBM, native collagen membrane and CTG applied to the buccal surface; (H) 12 months control, buccal view; and (I) 12 months control X‐ray.

**FIGURE 3:**
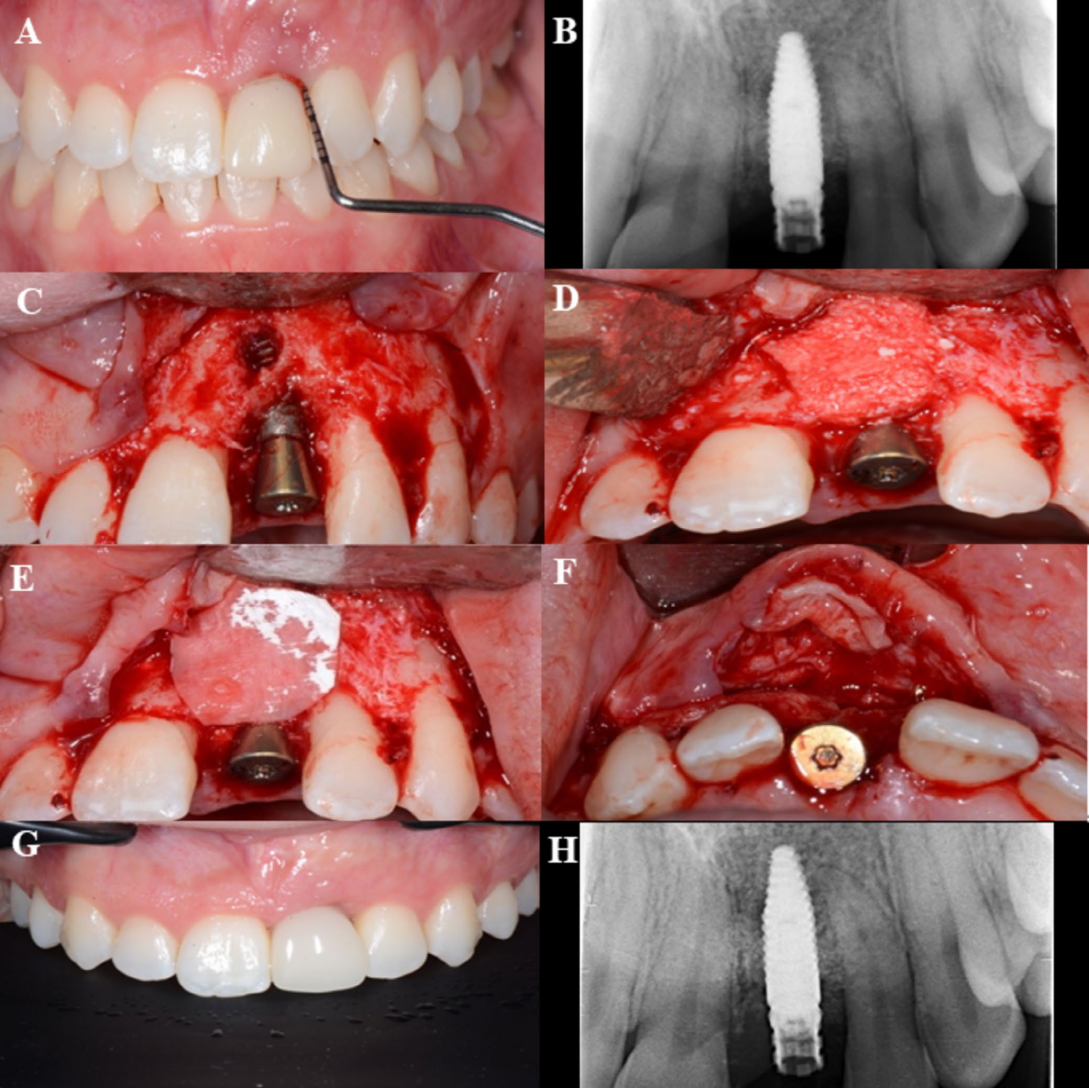
Test group, strata 2—case report, regio 21. (A) Baseline, buccal view; (B) baseline X‐ray; (C) surgery, flap elevation, buccal view of the peri‐implant bone defect; (D) buccal view of the implant with collagenated DBBM; (E) application of native collagen membrane; (F) CTG applied to the buccal flap; (G) 12 months control, buccal view; and (H) 12 months control X‐ray.

**FIGURE 4:**
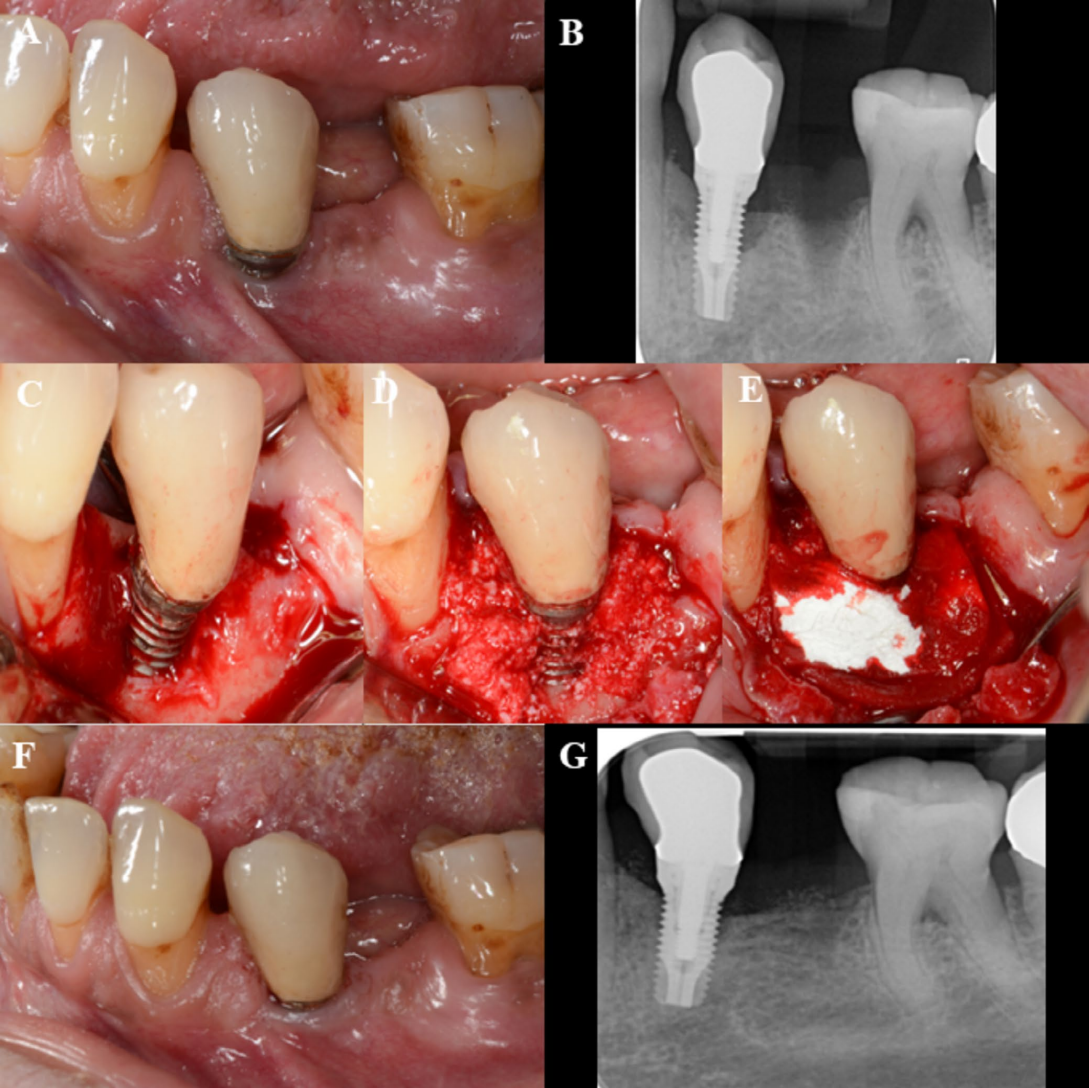
Control group, strata 1—case report, regio 45. (A) Baseline, buccal view; (B) baseline X‐ray; (C) surgery, flap elevation, buccal view of the peri‐implant bone defect; (D) buccal view of the implant with collagenated DBBM; (E) application of native collagen membrane; (F) 12 months control, buccal view; and (G) 12 months control X‐ray.

**FIGURE 5:**
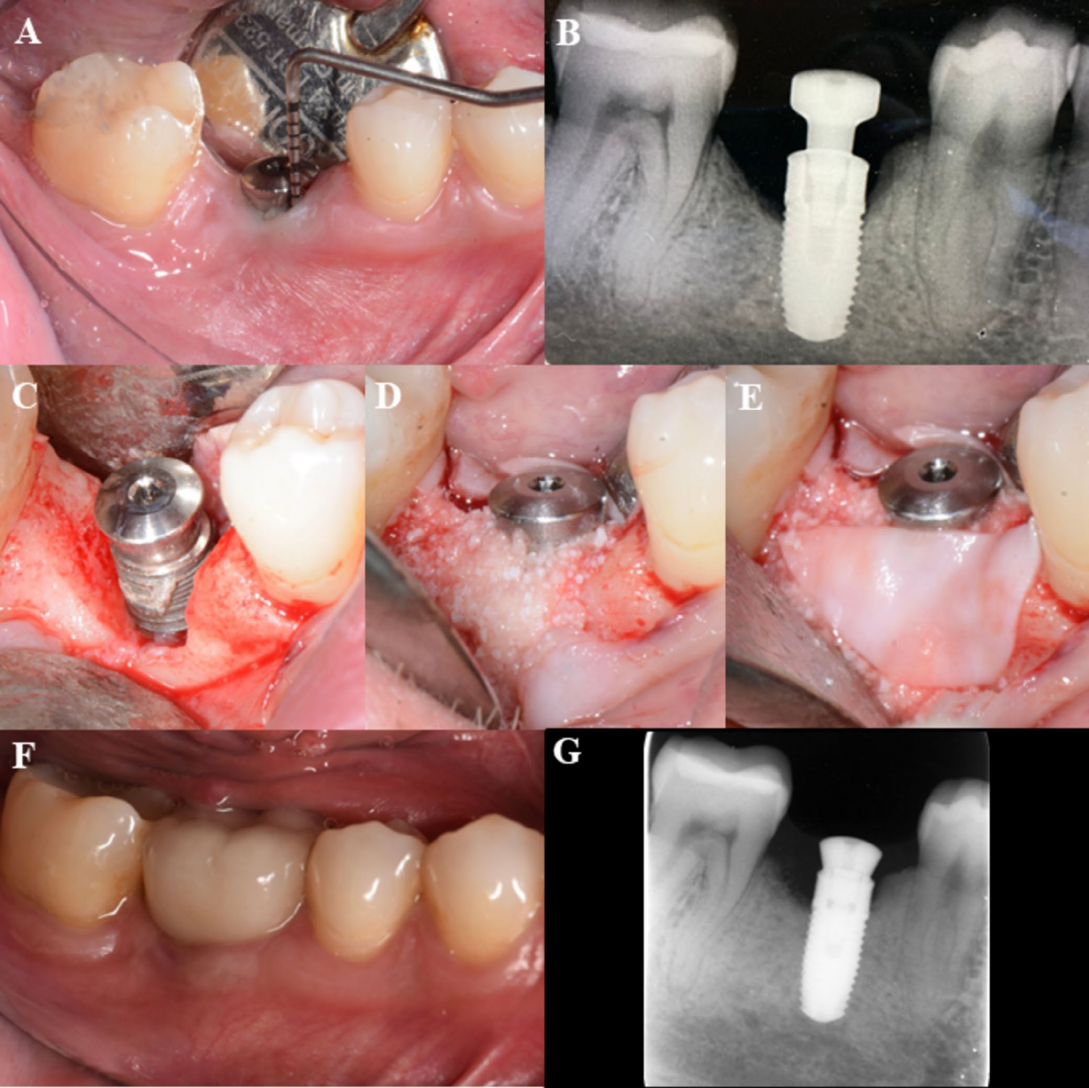
Control group, strata 2—case report, regio 46. (A) Baseline, buccal view; (B) baseline X‐ray; (C) surgery, flap elevation, buccal view of the peri‐implant bone defect; (D) buccal view of the implant with collagenated DBBM; (E) application of native collagen membrane; (F) 12 months control, buccal view; and (G) 12 months control X‐ray.

In total, 32 randomized patients were enrolled, treated, and completed the 12‐month follow‐up without any losses during the study period (Figure 1). At baseline, the test and control groups were broadly comparable in terms of socio‐demographic, systemic, and implant‐related variables. No statistically significant differences were found regarding the patients' characteristics (Table 1, Table S1). Implant‐specific parameters (e.g., jaw location, implant type, surface characteristics, prosthesis retention, etc.) were also homogeneously distributed (Table 2). Clinical cases are presented for the test group, strata 1 (Figure 2) and strata 2 (Figure 3), as well as for the control group, strata 1 (Figure 4) and strata 2 (Figure 5).

**TABLE 1:** Demographic data.

	Total (*N* = 32)	Test group (*N* = 16)	Control group (*N* = 16)
Gender (f/m)	16/16	7/9	9/7
Age (Mean ± SD)	56.9 ± 12.3	53.2 ± 13.5	60.6 ± 10.0
BMI (Mean ± SD)	23.3 ± 3.5	22.6 ± 3.6	23.9 ± 3.4
Smoking (yes/no; *N* (%))	9/23 (28.1/71.9)	5/11 (31.3/66.7)	4/12 (25/75)
Diabetes (yes/no; *N* (%))	1/31 (3.1/96.9)	0/16 (0/100)	1/15 (6.3/93.7)
Cardiovascular diseases (yes/no; *N* (%))	5/27 (15.6/84.4)	2/14 (12.5/87.5)	3/13 (18.8/81.2)
Hypertension (yes/no; *N* (%))	7/25 (21.9/78.1)	3/13 (18.8/81.2)	4/12 (25/75)
Myocardial infarction (yes/no; *N* (%))	1/31 (3.1/96.9)	0/16 (0/100)	1/15 (6.2/93.8)
Dyslipidemia (yes/no; *N* (%))	3/29 (9.4/90.6)	1/15 (6.2/93.8)	2/14 (12.5/87.5)
Stroke (yes/no; *N* (%))	1/31 (3.1/96.9)	0/16 (0/100)	1/15 (6.3/93.8)
Arthritis (yes/no; *N* (%))	3/29 (9.4/90.6)	3/13 (18.8/81.2)	0/16 (0/100)
Osteoporosis (yes/no; *N* (%))	2/30 (6.2/93.8)	1/15 (6.2/93.8)	1/15 (6.2/93.8)
Respiratory diseases (yes/no; *N* (%))	2/30 (6.2/93.8)	1/15 (6.3/93.7)	1/15 (6.3/93.7)
Asthma (yes/no; *N* (%))	1/31 (3.1/96.9)	1/15 (6.2/93.8)	0/16 (0/100)
Gastrointestinal diseases (yes/no; *N* (%))	3/29 (9.4/90.6)	2/14 (12.5/87.5)	1/15 (6.3/93.8)
Renal diseases (yes/no; *N* (%))	1/31 (3.1/96.9)	0/16 (0/100)	1/15 (6.3/93.8)
Depression (yes/no; *N* (%))	1/31 (3.1/96.9)	0/16 (0/100)	1/15 (6.2/93.8)
Medication intake (yes/no; *N* (%))	16/16 (50/50)	9/7 (56.2/43.8)	7/9 (43.8/56.2)

Abbreviations: %, percentage; f, female; m, male; *N*, number; SD, standard deviation.

**TABLE 2:** Implant‐specific parameters—descriptive statistics.

	Total (*N* = 32)	Test group (*N* = 16)	Control group (*n* = 16)
Jaw (maxilla/mandible)	15/17	8/8	7/9
Implant type (BL/TL; *N* (%))	20/12 (62.5/37.5)	11/5 (68.8/31.3)	9/7 (56.3/43.8)
Implant surface (rough/smooth; *N* (%))	30/2 (93.8/6.3)	15/1 (93.8/6.3)	15/1 (93.8/6.3)
Prosthesis type (C/B/O; *N* (%))	16/13/3 (50/40/9.4)	9/5/2 (56.3/31.2/12.5)	7/8/1 (43.8/50/6.3)
Prosthetic retention (CM/SR/RD; *N* (%))	24/4/4 (15/12.5/12.5)	10/3/3 (62.4/18.8/18.8)	14/1/1 (87.4/6.3/6.3)
Implants in function (years; Mean ± SD)	2.19 ± 0.82	2.19 ± 0.75	2.19 ± 0.91

Abbreviations: %, percentage; B, bridge; BL, bone level; C, crown; CM, cemented; N, number; O, overdenture; RD, removable denture; SD, standard deviation; SR, screw retained; TL, tissue level.

### 3.2 Primary Outcome—Clinical Attachment Level (CAL)

Both groups demonstrated a statistically significant gain in CAL from T_0_ to T_1_ (*p* = 0.003), which remained stable thereafter (T_1_‐T_2_, *p* > 0.05). Both groups exhibited significant CAL improvement over 12 months (*p* < 0.001), with the test group achieving a greater overall gain compared to the control (*p* = 0.022). The majority of improvement occurred by T_1_ and was maintained thereafter (T_2_) (Tables 3, 4, 5; Figure S1G).

**TABLE 3:** Mean ± standard deviation (SD) of clinical parameters at the implant level in test and control groups, with intergroup comparisons at T_1_ and T_2_ and overall time effect (T_0_–T_2_, Brunner‐Langer ATS).

Clinical parameters (Mean ± SD)	Group	T_1_	T_2_	*p T_0_‐T_2_
CAL	Test	3.91 ± 1.73	3.32 ± 0.85	***p* < 0.001**
	Control	4.11 ± 1.23	4.16 ± 1.32	
	Test vs. control	*p* = 1.000	*p* = 0.240	
PPD	Test	3.31 ± 0.78	3.05 ± 0.51	***p* < 0.001**
	Control	3.46 ± 0.92	3.59 ± 1.20	
	Test vs. control	*p* = 1.000	*p* = 0.762	
KMW	Test	3.38 ± 2.12	4.19 ± 3.06	***p* < 0.001**
	Control	2.73 ± 1.91	2.98 ± 2.05	
	Test vs. control	*p* = 1.000	*p* = 0.915	
REC	Test	1.00 ± 1.15	0.65 ± 1.01	***p* < 0.001**
	Control	0.81 ± 0.97	0.54 ± 0.73	
	Test vs. control	*p* = 1.000	*p* = 1.000	
MT	Test	3.21 ± 1.38	2.24 ± 0.97	***p* < 0.001**
	Control	2.28 ± 0.78	2.04 ± 0.95	
	Test vs. control	*p* = 0.141	*p* = 1.000	
BoP	Test	26.0 ± 30.3	24.0 ± 25.0	***p* < 0.001**
	Control	45.8 ± 35.2	27.2 ± 31.5	
	Test vs. control	*p* = 0.306	*p* = 1.000	
SUP	Test	1.00 ± 4.17	0.00 ± 0.00	***p* < 0.001**
	Control	12.5 ± 28.8	1.00 ± 4.17	
	Test vs. control	*p* = 1.000	*p* = 1.000	
PI	Test	17.7 ± 18.7	17.7 ± 21.5	*p* = 0.056
	Control	21.8 ± 22.5	28.2 ± 29.0	
	Test vs. control	*p* = 1.000	*p* = 1.000	

*Note:* *p value T_0_‐T_2_—Bruner‐Langer ATS test for time effect; intergroup comparisons—Mann–Whitney test with Bonferroni correction; significant *p*‐values (< 0.05) are indicated in bold.

Abbreviations: BoP, bleeding on probing (% of sites); CAL, clinical attachment level; KMW, keratinized mucosa width; MT, mucosal thickness; PI, plaque index (% of sites); PPD, pocket probing depth; REC, buccal recession; SUP, suppuration on probing (% of sites); T_0_, baseline; T_1_, 6 months; T_2_, 12 months.

### 3.3 Secondary Outcomes

Mean ± standard deviation values of all clinical parameters at the implant level, assessed at T_0_, T_1_, and T_2_, the overall time effect, as well as intergroup comparison, are presented in Tables 3 and 5. Intragroup comparisons are shown in Table 4.

**TABLE 4:** Results of the intragroup comparison between T_0_, T_1_, and T_2_. Differences T_1_‐T_0_, T_1_‐T_2_, and T_0_‐T_2_ expressed as mean ± SD.

Clinical parameters (*p* values)	Group	T_0_ vs. T_1_		T_1_ vs. T_2_		T_0_ vs. T_2_	
		Mean ± SD	*p*	Mean ± SD	*p*	Mean ± SD	*p*
CAL	Test	−2.63 ± 2.25	**0.003**	−0.58 ± 1.37	0.335	−3.21 ± 1.57	**< 0.001**
	Control	−1.69 ± 1.24	**0.003**	0.04 ± 0.77	1.000	−1.65 ± 1.28	**0.048**
PPD	Test	−2.99 ± 1.68	**0.003**	−0.26 ± 0.54	0.336	−3.25 ± 1.59	**< 0.001**
	Control	−2.10 ± 1.22	**0.003**	0.14 ± 0.80	1.000	−1.97 ± 1.23	**< 0.001**
KMW	Test	1.44 ± 1.98	**0.012**	0.81 ± 1.20	0.009	2.25 ± 2.89	**0.003**
	Control	0.01 ± 1.62	1.000	0.25 ± 1.06	1.000	0.26 ± 1.49	1.000
REC	Test	0.56 ± 0.94	0.084	−0.32 ± 0.76	0.147	0.24 ± 0.49	0.237
	Control	0.43 ± 0.67	0.054	−0.06 ± 0.33	1.000	0.36 ± 0.63	0.123
MT	Test	1.17 ± 1.35	**0.027**	−0.97 ± 1.02	**0.012**	0.29 ± 1.47	1.000
	Control	0.58 ± 1.28	0.186	−0.24 ± 0.62	0.415	0.34 ± 1.11	0.447
BoP	Test	−61.5 ± 42.5	**0.006**	−2.17 ± 24.2	1.000	−63.5 ± 34.0	**0.003**
	Control	−32.3 ± 44.8	0.054	−18.8 ± 39.8	0.291	−51.0 ± 42.3	**0.003**
SUP	Test	−16.7 ± 22.0	**0.048**	−1.00 ± 4.17	0.951	−17.7 ± 23.2	**0.042**
	Control	−17.7 ± 47.3	0.510	−11.5 ± 29.7	0.423	−29.2 ± 41.0	**0.048**
PI	Test	−26.0 ± 43.8	0.105	0.00 ± 30.5	1.000	−26.0 ± 38.0	0.057
	Control	−18.8 ± 49.8	0.612	6.33 ± 23.5	0.831	−12.5 ± 58.8	1.000

*Note:* Wilcoxon test and Bonferroni correction, significant *p*‐values (< 0.05) are indicated in bold.

Abbreviations: BoP, bleeding on probing; CAL, clinical attachment level; KMW, keratinized mucosa width; MT, mucosal thickness; PI, plaque index; PPD, pocket probing depth; REC, buccal recession; SUP, suppuration on probing; T_0_, baseline; T_1_, 6 months; T_2_, 12 months.

**TABLE 5:** Intergroup comparison of primary and secondary outcomes between the test and the control groups from T_0_ to T_2_.

Outcomes	Group	Mean ± SD	*p*
CAL	Test	−3.21 ± 1.57	**0.022**
	Control	−1.65 ± 1.28	
PPD	Test	−3.25 ± 1.59	0.052
	Control	−1.97 ± 1.23	
KMW	Test	2.25 ± 2.89	**0.010**
	Control	0.26 ± 1.49	
REC	Test	0.36 ± 0.63	0.677
	Control	0.29 ± 1.47	
MT	Test	0.29 ± 1.47	0.673
	Control	0.34 ± 1.11	
BoP	Test	−63.5 ± 34.0	0.103
	Control	−51.0 ± 42.3	
SUP	Test	−17.7 ± 23.2	0.777
	Control	−29.2 ± 41.0	
PI	Test	−26.0 ± 38.0	0.541
	Control	−12.5 ± 58.8	

*Note:* Time–group interaction effect (Brunner–Langer ATS), significant for *p*‐value < 0.05, indicated in bold.

Abbreviation: SD, standard deviation.

**TABLE 6:** Mean bone level.

			Test group (*N* = 32)	Control group (*N* = 32)	Test vs. control **p
Mean Bone Level	T_0_	Mean ± SD	3.72 ± 1.49	2.92 ± 1.30	0.188
	T_2_	Mean ± SD	1.05 ± 0.90	1.63 ± 1.19	0.320
	T_0_ vs. T_2_	*p value	**< 0.001**	**0.001**	

*Note:* *p value—intragroup comparison, Wilcoxon's test and Bonferroni's correction, significant at *p* < 0.05; **p value—intergroup comparison, Mann–Whitney test and Bonferroni's correction, significant at *p* < 0.05. Significant *p*‐values are indicated in bold.

Abbreviations: *N*, number; SD, standard deviation; T_0_, baseline; T_2_, 12 months.

#### 3.3.1 Probing Pocket Depth (PPD)

Both the test and control groups exhibited a significant reduction in PPD from T_0_ to T_1_ (*p* = 0.003), which was maintained through T_2_. Although both groups followed a similar trend over the 12‐month period, the overall time‐dependent reduction was statistically significant in both groups (*p* < 0.001). Although the difference between groups did not reach statistical significance (*p* = 0.052), the test group showed a numerically greater improvement (See Figure S1B).

#### 3.3.2 Keratinized Mucosa Width (KMW)

In the test group, KMW increased markedly over time (*p* < 0.001), with improvements evident from T_0_ to T_1_ (*p* = 0.012) and further increases by T_2_ (*p* = 0.009). In contrast, the control group showed no significant change throughout the study period (*p* = 1.000). The overall time‐dependent pattern differed significantly between groups (*p* = 0.010), although intergroup comparison at T_2_ revealed no significant differences (*p* = 0.915) (See Figure S1C).

#### 3.3.3 Mucosal Recession (REC) and Mucosal Thickness (MT)

For both groups, REC showed a statistically significant overall increase from T_0_ to T_2_ (*p* < 0.001), but neither intragroup nor intergroup comparisons at specific time points reached significance (*p* > 0.05) (See Figure S1D).

MT increased significantly in the test group from T_0_ to T_1_ (*p* = 0.027), followed by a significant reduction from T_1_ to T_2_ (*p* = 0.012), while the control group remained stable across all intervals (*p* > 0.05). No significant differences were observed between groups at any time point (See Figure S1E).

#### 3.3.4 Bleeding on Probing (BoP)

BoP decreased significantly in the test group from T_0_ to T_1_ (*p* = 0.006) and remained stable thereafter (T_1_‐T_2_; *p* > 0.05). The control group showed a similar trend, but the initial reduction did not reach statistical significance (*p* = 0.054). Over 12 months, both groups demonstrated significant overall decreases (*p* = 0.003), with no significant differences between them (See Figure S1F).

#### 3.3.5 Suppuration (SUP)

The number of sites with suppuration significantly decreased over time (T_0_‐T_2_; *p* < 0.001), with a similar pattern across all three timepoints in both groups (*p* = 0.777). In the test group, the reduction was already significant between T_0_ and T_1_ (*p* = 0.048), while in the control group, it became significant by T_2_ (*p* = 0.048). No significant intergroup differences were detected at any time point (*p* > 0.05) (See Figure S1G).

#### 3.3.6 O'Leary Plaque Index (PI)

A decreasing trend in PI was noted in both groups over time. However, the reductions from T_0_ did not reach statistical significance at either T_1_ or T_2_ (*p* > 0.05). In the test group, the change at 12 months approached significance (*p* = 0.057), while no between‐group differences were observed (See Figure S1H).

#### 3.3.7 Radiographic Marginal Bone Level (BL)

Both groups showed a significant bone gain from T_0_ to T_2_ (*p* < 0.001). Intragroup analyses confirmed significant improvements in the test (*p* < 0.001) and control (*p* = 0.001) groups, while no significant intergroup differences were observed at either time point (*p* = 0.188 and *p* = 0.320, respectively). The overall time‐group interaction was significant (*p* < 0.001), indicating that the extent of bone gain differed between groups (Table 6; See Figure S2).

#### 3.3.8 Peri‐Implant Bone Defects Morphology (Monje et al. 2019)

According to the classification proposed by Monje et al. (2019), most defects were categorized as Class IIIc, followed by Classes IIIb and Ic, while Classes Ia, Ib, and IIIa were less frequent. No statistically significant differences in defect distribution were found between groups, either in the overall sample or within strata (*p* = 0.697). Logistic regression analysis further showed that defect morphology was not significantly associated with the likelihood of disease resolution at 12 months, whether defined by the EFP criteria (overall *p* = 0.403; OR = 0.50, 95% CI 0.07–3.45, *p* = 0.482) or by Derks et al. (*p* = 0.312; OR = 0.40, 95% CI 0.06–2.77, *p* = 0.353).

#### 3.3.9 Disease Resolution According to EFP Criteria

Disease resolution, following EFP criteria, was achieved in 62.5% of implants in the test group and 56.3% in the control group (difference not significant; OR = 1.30, 95% CI: 0.32–5.33; *p* = 0.719). Stratified analysis revealed similar nonsignificant differences across KM strata (Stratum 1: 57.1% vs. 50.0%, *p* = 0.782; Stratum 2: 66.7% vs. 62.5%, *p* = 0.858). The group‐by‐stratum interaction was also nonsignificant (*p* = 0.942), indicating a consistent treatment effect across KM levels. See Table S2 and Figure S3.

#### 3.3.10 Disease Resolution According to Derks et al. Criteria

Following the approach of Derks et al., the disease resolution was 62.5% in the test group and 50.0% in the control group (difference not significant; OR = 1.67, 95% CI: 0.41–6.82; *p* = 0.477). Within stratum 1, disease resolution occurred in 57.1% of test and 37.5% of control implants (OR = 2.22; *p* = 0.450), and in stratum 2, 66.7% and 62.5%, respectively (OR = 1.20; *p* = 0.858). The interaction between group and KM stratum was also nonsignificant (*p* = 0.674). Descriptive data are reported in Table S2 and Figure S4.

#### 3.3.11 Early Wound Healing

Healing was assessed at one and 2 weeks postoperatively using the Early Wound Healing Index (EHI; Wachtel et al. 2003; score range 1 = complete flap closure without inflammation to 5 = incomplete closure with pronounced inflammation), recorded by the surgeon. At 1 week, most sites presented with an EHI score of 1 (37.5% in both groups) or 2 (50.0% in the test group, 43.8% in the control group), indicating complete closure with none or only slight residual inflammation. Higher EHI scores (≥ 3) were rare (< 13% in either group). At 2 weeks, the large majority of sites achieved an EHI score of 1 (68.8% test, 75.0% control), with the remainder showing only mild erythema (score 2) or, in isolated cases, a score of 3. No sites exhibited EHI scores of 4 or 5 at this stage. No statistically significant differences were observed between groups at either time point in the overall sample or within strata (*p* > 0.05). No postoperative complications such as suppuration, necrosis, or membrane exposure were recorded.

#### 3.3.12 Surgical Time

The mean surgical duration for the overall sample was 68.5 ± 26.0 min in the test group and 60.3 ± 15.8 min in the control group (median (IQR): 72.5 (53.0–87.0) vs. 60.0 (47.0–70.0) min, respectively), with no statistically significant difference (*p* > 0.05). In strata 1, however, surgical time was significantly longer in the test group (*p* = 0.040), possibly reflecting greater procedural complexity due to connective tissue grafting. In strata 2, surgical times were comparable between groups (*p* > 0.05). Across all strata, recorded durations ranged from 40 to 104 min.

## 4 Discussion

Reconstructive therapy for the treatment of peri‐implantitis is considered safe and effective with low rates of implant failure in the short‐ to mid‐term (Solderer et al. 2024). In terms of the success rates of reconstructive peri‐implantitis surgical treatment, the results of the present study are in line with the literature. Derks et al. (2022) reported a composite success rate of 16.4% for reconstructive treatment, whereas in the present study rates of 62.5% and 50% were observed for test and control groups, respectively. A possible explanation for the higher rates reported in the present study could relate to the baseline PPD values, which were lower in our study (6.3 and 5.6 mm on average in the test and control groups, respectively) compared to those reported by Derks et al. (2022) (8.6 mm average). Similarly, the baseline MBL was inferior in our study 3.72 and 2.92 in test/control groups versus 6.1 mm in the study by Derks et al. (2022), suggesting less advanced forms of disease at baseline in the present cohort. The importance of PPD at baseline on further disease progression has been confirmed (Karlsson et al. 2019). Furthermore, differences in decontamination protocol (use of erythritol powder in the present study design) could explain in part the different results. Success rates in this study are somewhat more similar to those reported by Monje et al. (2022): the authors reported 77.1% disease resolution, while we achieved 62.5% and 56.3% (respectively, test and control groups with EFP criteria definition). The surgical and decontamination protocols are, however, different from those employed in the present study (hydrogen peroxide rinses for decontamination, use of an allograft, and implantoplasty in uncontained defect components) and could potentially account at least in part for the different rates of disease resolution. Overall, the results of the present study suggest that a reconstructive approach for the treatment of peri‐implantitis can lead to success rates at 12 months that are in line with those from other studies, regardless of the use of an adjunctive CTG.

In the present study, no difference in MBL could be detected between groups, and in both test and control groups, there were significant reductions in bone level (meaning gain in radiographic defect fill) between T_0_ and T_2_. Derks et al. (2022) demonstrated 22.2% of cases with pronounced MBL gain of > 2 mm when a bone graft was applied in the reconstructive treatment of intrabony defects. Additionally, they noted the MBL improvement at 12 months of about 1 mm, which is quite similar to the changes found in our study within the control group, slightly inferior to the improvements reported within the first year for the test group.

Our results demonstrated that CAL gain was significant within both groups, and the intergroup difference at 12 months was statistically significant, in line with data reported from other studies on reconstructive treatment (Schwarz et al. 2010). This is a noteworthy result, as the change in CAL was the primary outcome of the present study, although it is not clear at this point whether the difference between the test and control groups (of approximately 1.5 mm at 12 months) is clinically significant. Nevertheless, the change observed in the test group at 12 months is approximately twice that of the control group and exceeds the changes reported in other studies evaluating CAL at the same time point (Schwarz et al. 2011). Additionally, whether this parameter remains stable and the difference between groups is maintained beyond the 12‐month time‐point cannot be answered by the present dataset. Nevertheless, it is the author's feeling that the greater gain in CAL in the test group suggests that both REC and especially PPD changes were more pronounced in the test group, and this might also translate into a long‐term clinically more meaningful difference between treatment arms.

In implant dentistry, the role of peri‐implant soft tissues in implant health maintenance has been documented and elaborated through the literature (Zigdon and Machtei 2008; Thoma et al. 2022). The results of the present study suggested that the soft tissue graft yielded a clinical increase in KM (though not confirmed with histological data) in the test group from baseline to the 12‐month time point, with a 2.25 ± 2.89 increment at 12 months. On the other hand, there was a clinically negligible change in the control group amounting to 0.26 ± 1.49, indicating a difference of more than 8‐fold between the two groups. This represents a clinically meaningful gain in the test group, similar to what can be achieved when performing a keratinized tissue augmentation by means of an autologous free‐gingival graft (Soldini et al. 2025), though it remains to be clarified whether this difference will be maintained over time.

Indeed, when treating gingival recessions around teeth, Cairo (Cairo et al. 2014) reported a higher width of keratinized tissue at 12 months in the CTG group versus the control, which was treated with a coronally advanced flap alone. A similar finding was reported for immediate implant placement (Guglielmi et al. 2022). On the other hand, studies on reconstructive treatment with no simultaneous soft tissue grafting have reported stable levels of KM or even a slight loss at 1 year (Roccuzzo et al. 2021; Derks et al. 2022). The speculated increased keratinization over time could be beneficial for peri‐implant health and improve the prognosis of the treated implants over time. Even though KM is regarded as an important factor in maintaining peri‐implant health and has been linked to better plaque control and patient comfort (Stefanini et al. 2023), its specific effect on the surgical resolution of peri‐implantitis remains uncertain. In this study, no statistically significant difference in treatment success rates was found between groups, which may reflect the complex nature of peri‐implantitis healing, the impact of other surgical factors, or the short follow‐up time. Nonetheless, KM augmentation may still help achieve long‐term tissue stability and make maintenance easier (Thoma et al. 2018). Differences between the two groups may thus appear with longer observation periods.

Furthermore, when interpreting these findings, it should be noted that the measurement of KMW could be prone to some error, as the roll technique was employed without adjunctive aids such as iodine staining. While the roll test is widely used and has been validated against staining techniques in clinical studies, showing no significant intermethod differences (Lang and Löe 1972; Guglielmoni et al. 2001), staining can provide a clearer demarcation of the mucogingival junction and may improve measurement precision, especially over serial assessments (Bhatia et al. 2015). Additionally, in the absence of histologic verification, the precise nature of the newly formed peri‐implant soft tissue cannot be confirmed, as histologic sampling is not feasible in this clinical trial context. Therefore, the reported increases in keratinized mucosa width and mucosal thickness should be interpreted cautiously, as they do not establish definitive tissue composition or maturation (Berglundh and Lindhe 1996). Furthermore, the relatively small sample size and short follow‐up time of the present sample need to be kept in mind when interpreting clinically measured gains in KMW. Still, the clinical stability of the obtained soft tissues presents an important parameter for the evaluation of treatment success (Sanz et al. 2022).

In our study, both groups displayed an increased MT over time, with significant differences between the two groups. The tendency for grafted sites to display an increase in thickness over time has been reported by other studies (Thoma et al. 2022) that have used similar methods of evaluating MT, though the 12‐month values of thickness are lower in our study compared to the study by Thoma (2.24 ± 0.97 vs. 3.1 ± 1.3 mm). Possibly, the harvesting technique (de‐FGG versus single‐incision palatal graft) could explain the differences, as well as the measurement location (measured at 1 mm from the margin by Thoma and 2 mm in our study). Increased MT could possibly lead to greater bone stability over time (Puisys and Linkevicius 2013; Di Gianfilippo et al. 2020), and there is evidence to support the concept that thicker buccal mucosa is protective of crestal peri‐implant bone levels (Kaminaka et al. 2015). The protective effect of a thicker mucosa on bone levels needs to be explored over a longer period of time to determine whether it could also be true in the present sample, as overall interproximal bone levels did not differ among groups at 12 months.

REC values for both groups in the present study are similar to those reported in other studies on reconstructive therapy (Roccuzzo et al. 2021; Derks et al. 2022; Soldini et al. 2025). No significant differences were found between groups in buccal REC, which is somewhat surprising. In both groups in the present study, REC appeared to decrease between 6 and 12 months at a similar rate. In a case series conducted by Schwarz et al. (2014), there was a slight increase in recession (0.46 ± 0.77 mm) up to 6 months after combined therapy with soft tissue augmentation, however, data were not collected long‐term. The reason for the lack of difference among groups is not well understood, however, it could potentially be related to the relatively “apical” position of the graft on the buccal aspect. It is possible that positioning the graft in a “poncho” configuration around the implant collar might have limited REC in the most coronal portion of the flap to a greater extent. Nonetheless, the buccal REC appears very contained at 12 months (< 1 mm in both groups), similarly to results reported by other researchers (Soldini et al. 2025; Derks et al. 2022).

Although adding a connective tissue graft did not lead to a statistically significant improvement in disease resolution rates at 12 months between groups, the observed KMW and MT increases may still be clinically important. Better peri‐implant soft tissue conditions are linked to improved plaque control, increased patient comfort during oral hygiene, and possibly greater long‐term stability of treatment results, which could become evident beyond the initial follow‐up period (Perussolo et al. 2018; Ramanauskaite et al. 2022).

Furthermore, baseline peri‐implant bone defects presented a range of morphologies, most frequently two‐ and three‐wall defects, which are generally considered to offer more favorable healing potential than shallow or horizontal configurations (Monje et al. 2019; Aghazadeh et al. 2020). While such factors can influence treatment outcomes, both groups in our study exhibited a similar distribution of defect types, suggesting that differences in healing cannot be attributed solely to baseline defect morphology.

Regarding treatment duration, no significant differences were found between treatment groups. Within Strata 1, however, the duration of test treatment was significantly longer than control treatment. This could indicate a more difficult surgical management, especially in the most coronal portion of the flap, and graft fixation to the inside of the flap in the presence of minimal/absent KM. No differences were present within strata 2 between groups. The decontamination protocol used in the present study includes a combination of titanium brushes, air‐powder system, and sterile saline rinses. The use of air‐powder systems has been proposed in the context of resective (Luengo et al. 2022; Hentenaar et al. 2022; Lasserre et al. 2020; Toma et al. 2019) and reconstructive (Friedmann et al. 2024) surgical treatment of peri‐implantitis, despite reports of undesired effects with open flap use (Bassetti et al. 2014). Overall, the success rates reported in the present sample suggest that reconstructive therapy performed with collagenated demineralized bovine bone and a native collagen membrane in combination with mechanical/physical (titanium curettes, titanium brushes, erythritol powder) and chemical (saline rinses) decontamination may be considered a predictable option under the conditions tested in this study.

Limitations of the present study include its relatively small sample size and a 12‐month follow‐up period, which may not fully reflect the long‐term stability of peri‐implantitis treatment outcomes. The relatively small final cohort size (*n* = 32) may have limited the statistical power to detect certain intergroup differences, particularly for outcomes where trends favored the test group but did not reach statistical significance. For example, although the difference in PPD reduction between groups narrowly missed statistical significance (*p* = 0.052), the mean reduction was 94.5% greater in the test group compared to the control group (−3.21 mm vs. −1.65 mm), representing a clinically relevant effect that might have reached significance with a larger sample size. In contrast, the difference in disease resolution rates according to the EFP criterion (62.5% vs. 56.3%) was small and below the threshold generally considered clinically relevant, making it unlikely that an increased sample size would have altered this outcome. However, many surgical peri‐implantitis studies initially report outcomes at 12 months (Soldini et al. 2025; Derks et al. 2022; İnce Kuka and Gürsoy 2024; Regidor et al. 2023; González et al. 2021), with some subsequently presenting longer‐term follow‐up data (Roccuzzo et al. 2017; Romeo et al. 2007). This patient cohort will continue to be monitored, and the results of the 24‐month follow‐up will be reported in a future publication. Furthermore, the lack of histological data prevents reaching definitive conclusions in merit to the keratinization of the buccal flap tissue induced by the underlying sCTG. Finally, if superimposition of STL and DICOM files had been available, they could have provided greater insight into the exact dynamics of MT changes over time.

## 5 Conclusion

In conclusion, the results of the present multicenter RCT suggest that reconstructive treatment of peri‐implantitis may be a predictable approach, yielding significant improvements in clinical and radiographic parameters at 12 months. The addition of an sCTG does not appear to impact disease resolution. Consequently, the clinical benefit of sCTG addition needs to be further explored, given the clinical impact of Disease Resolution. However, adding sCTG to reconstructive peri‐implantitis treatment increases CAL gain and improves MT and KMW. The long‐term effects of sCTG augmentation remain unknown and should be further investigated to determine whether this adjunctive treatment provides long‐term benefits for patients undergoing peri‐implantitis therapy.

## Author Contributions

L.P.H. and I.M. conceived the study, developed the methodology, performed the surgical procedures, and contributed to manuscript writing. M.V. curated the data, conducted clinical examinations, interpreted the results, and participated in manuscript writing. C.M. was responsible for data collection and patient management. M.C. contributed to data collection and archiving and to writing – review and editing the manuscript. A.S. performed the radiographic analysis and reviewed the manuscript. L.C. and Z.A. supervised the study and manuscript preparation and approved the final version of the draft.

## Funding

This study was supported by an Osteology Foundation Young Research Grant (18‐117).

## Ethics Statement

This study was conducted in accordance with the Declaration of Helsinki, ISO EN 14155, and applicable national legal and regulatory requirements. Ethical approval was obtained from the Comitato Etico Università “La Sapienza” (Approval No. 6109) and the Ethics Committee of the School of Dental Medicine, University of Belgrade (Approval No. 36/9, April 20, 2020).

## Conflicts of Interest

The authors declare no conflicts of interest.

## Supporting information


**Data S1:** CONSORT 2010 checklist of information to include when reporting a randomised trial*

**Figure S1:** Boxplots of clinical parameters at the implant level in test and control groups across time points (T_0_, T_1_, and T_2_). Each boxplot presents the distribution of observed values for clinical parameters: (A) clinical attachment level (CAL), (B) probing pocket depth (PPD), (C) keratinized mucosa width (KMW), (D) buccal recession (REC), (E) mucosal thickness (MT), (F) bleeding on probing (BoP), (G) suppuration (SUP), and (H) plaque index (PI).

**Figure S2:** Mean bone level (BL) changes over time in the test and control groups. (A) Boxplot showing mean bone levels at baseline (T_0_) and 12 months (T_2_); both groups showed bone gain over time (lower values represent bone level gain). (B) Relative effects plot from the Bruner‐Langer model, illustrating a more pronounced increase in the test group (lower values represent bone level gain).

**Figure S3:** Forest plot of odds ratios (ORs) with 95% confidence intervals for disease resolution between test and control groups, overall and stratified. Dots represent ORs; horizontal lines indicate 95% confidence intervals. The red dashed line marks the null value (OR = 1.0). None of the differences were statistically significant.

**Figure S4:** Forest plot of odds ratios (ORs) with 95% confidence intervals for disease resolution between test and control groups, overall and stratified, according to Derks et al. criteria. Dots represent ORs; horizontal lines indicate 95% confidence intervals. The red dashed line marks the null value (OR = 1.0). No statistically significant differences were observed across comparisons.

**Table S1:** Specific medication intake.

**Table S2:** Composite outcomes according to EFP and Derks et al.—descriptive statistics.

## Data Availability

The data that support the findings of this study are available from the corresponding author upon reasonable request.
