# Supplementary figures and images for: Effect of Connective Tissue Graft as an Adjunct to Guided Bone Regeneration in the Surgical Treatment of Peri‐Implantitis: A Dual‐Center Randomized Controlled Trial
Source: Clin Oral Implants Res. 2026 Jan 31;37(4):478–95. doi: 10.1111/clr.70093 (PMC13051417; doi:10.1111/clr.70093)

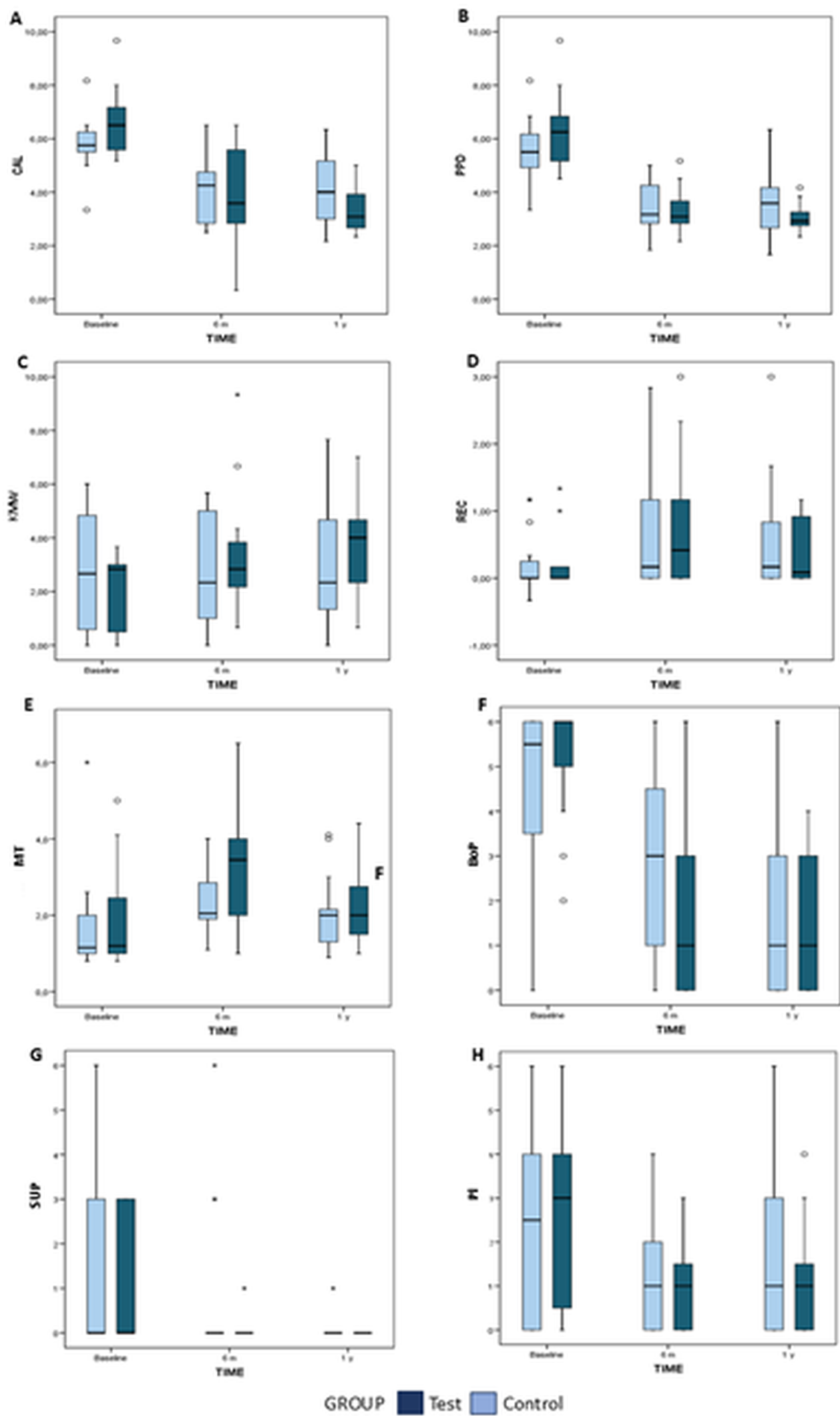

Supplement: Supplementary file 2 — Figure S1: Boxplots of clinical parameters at the implant level in test and control groups across time points (T0, T1, and T2). Each boxplot presents the distribution of observed values for clinical parameters: (A) clinical attachment level (CAL), (B) probing pocket depth (PPD), (C) keratinized mucosa width (KMW), (D) buccal recession (REC), (E) mucosal thickness (MT), (F) bleeding on probing (BoP), (G) suppuration (SUP), and (H) plaque index (PI). [file CLR-37-478-s006.pdf]

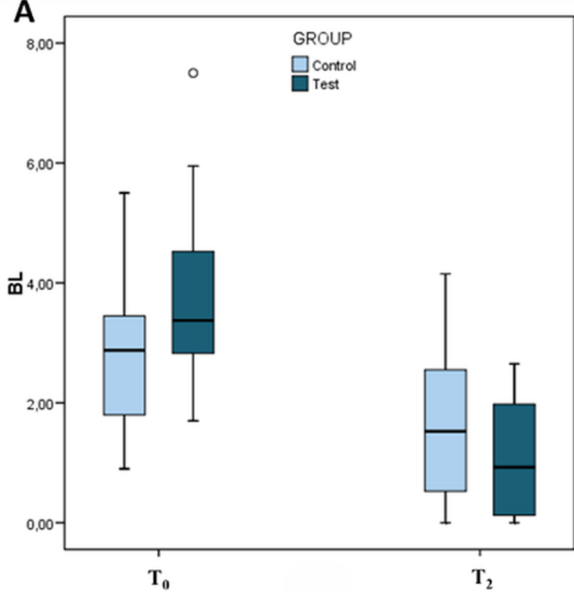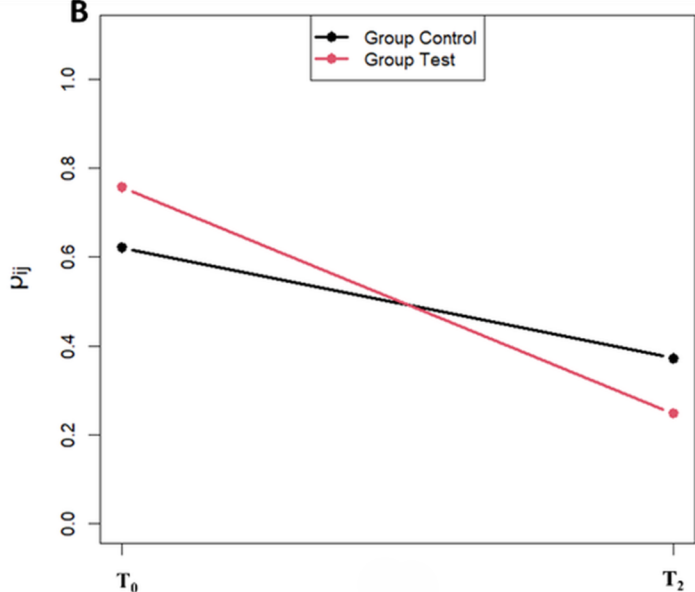

Supplement: Supplementary file 3 — Figure S2: Mean bone level (BL) changes over time in the test and control groups. (A) Boxplot showing mean bone levels at baseline (T0) and 12 months (T2); both groups showed bone gain over time (lower values represent bone level gain). (B) Relative effects plot from the Bruner‐Langer model, illustrating a more pronounced increase in the test group (lower values represent bone level gain). [file CLR-37-478-s003.pdf]

# Forest plot: EFP Disease Resolution criteria

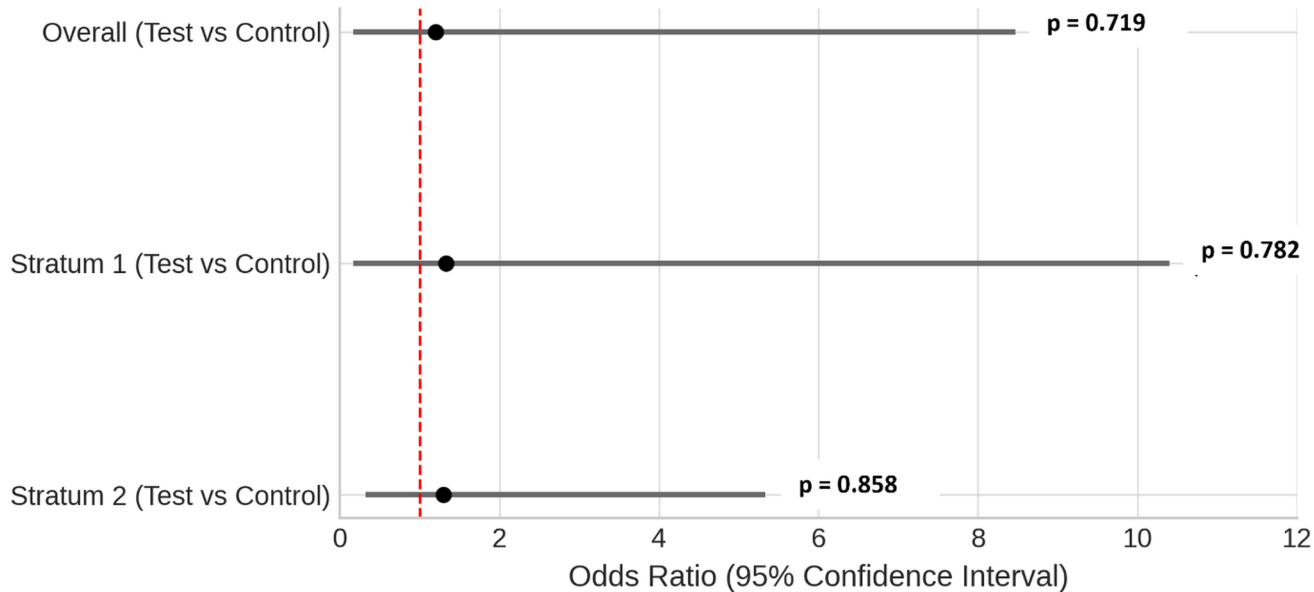

Supplement: Supplementary file 4 — Figure S3: Forest plot of odds ratios (ORs) with 95% confidence intervals for disease resolution between test and control groups, overall and stratified. Dots represent ORs; horizontal lines indicate 95% confidence intervals. The red dashed line marks the null value (OR = 1.0). None of the differences were statistically significant. [file CLR-37-478-s002.pdf]

## Forest plot: Derks et al. Disease Resolution criteria

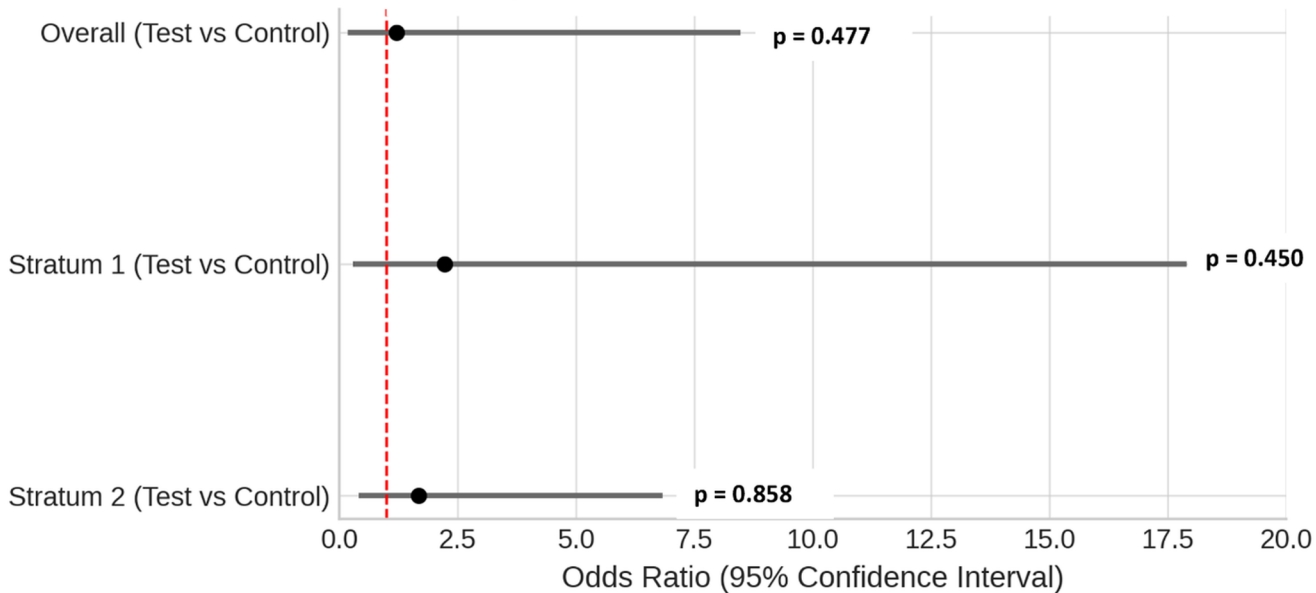

Supplement: Supplementary file 5 — Figure S4: Forest plot of odds ratios (ORs) with 95% confidence intervals for disease resolution between test and control groups, overall and stratified, according to Derks et al. criteria. Dots represent ORs; horizontal lines indicate 95% confidence intervals. The red dashed line marks the null value (OR = 1.0). No statistically significant differences were observed across comparisons. [file CLR-37-478-s007.pdf]
