# Supplementary material for: Effect of Connective Tissue Graft as an Adjunct to Guided Bone Regeneration in the Surgical Treatment of Peri‐Implantitis: A Dual‐Center Randomized Controlled Trial
Source: Clin Oral Implants Res. 2026 Jan 31;37(4):478–95. doi: 10.1111/clr.70093 (PMC13051417; doi:10.1111/clr.70093)
Supplement: Supplementary file 6 — Table S1: Specific medication intake. [file CLR-37-478-s005.docx]

Table S1 – Specific medication intake.

|  | Total  (N = 32) | Test group  (N = 16) | Control group  (N = 16) |
| --- | --- | --- | --- |
| Corticosteroids  (yes/no; N (%)) | 1/31 (3.1/96.9) | 1/15 (6.2/93.8) | 0/16 (0/100) |
| Antiplatelet drugs  (yes/no; N (%)) | 1/31 (3.1/96.9) | 1/15 (6.2/93.8) | 0/16 (0/100) |
| Antihypertensives  (yes/no; N (%)) | 9/23 (28.1/71.9) | 5/11 (31.2/68.8) | 4/12 (25/75) |
| Anticoagulants  (yes/no; N (%)) | 2/30 (6.2/93.8) | 1/15 (6.2/93.8) | 1/15 (6.2/93.8) |
| Hypolipidemic agents  (yes/no; N (%)) | 3/29 (9.4/90.6) | 2/14 (12.5/87.5) | 1/15 (6.2/93.8) |
| Thyroid drugs  (yes/no; N (%)) | 2/30 (6.2/93.8) | 1/15 (6.2/93.8) | 1/15 (6.2/93.8) |
| Proton pump inhibitors  (yes/no; N (%)) | 1/31 (3.1/96.9) | 1/15 (6.2/93.8) | 0/16 (0/100) |
| Vitamin D  (yes/no; N (%)) | 4/28 (12.5/87.5) | 2/14 (12.5/87.5) | 2/14 (12.5/87.5) |
| Antiepileptic drugs  (yes/no; N (%)) | 3/29 (9.4/90.6) | 2/14 (12.5/87.5) | 1/15 (6.2/93.8) |
| Antipsychotic drugs  (yes/no; N (%)) | 2/30 (6.2/93.8) | 1/15 (6.2/93.8) | 1/15 (6.2/93.8) |
| Other medication  (yes/no; N (%)) | 9/23 (28.1/71.9) | 6/10 (37.5/62.5) | 3/13 (12/8/81.2) |

N – number; f – female; m – male; SD – standard deviation; % - percentage.
