# Supplementary material for: Effect of Connective Tissue Graft as an Adjunct to Guided Bone Regeneration in the Surgical Treatment of Peri‐Implantitis: A Dual‐Center Randomized Controlled Trial
Source: Clin Oral Implants Res. 2026 Jan 31;37(4):478–95. doi: 10.1111/clr.70093 (PMC13051417; doi:10.1111/clr.70093)
Supplement: Supplementary file 7 — Table S2: Composite outcomes according to EFP and Derks et al.—descriptive statistics. [file CLR-37-478-s004.docx]

Table S2 – Composite outcomes according to EFP and Derks et al. – descriptive statistics

|  | | **STRATA** | | | | | | | | | | | | | | | | | | |
| --- | --- | --- | --- | --- | --- | --- | --- | --- | --- | --- | --- | --- | --- | --- | --- | --- | --- | --- | --- | --- |
|  |  | **TOTAL** | | | | | | **1** | | | | | | **2** | | | | | | |
|  |  | **Group** | | | | | | **Group** | | | | | | **Group** | | | | | | |
|  |  | **Total** | | **Test** | | **Control** | | **Total** | | **Test** | | **Control** | | **Total** | | **Test** | | **Control** | |  |
|  |  | **N** | **%** | **N** | **%** | **N** | **%** | **N** | **%** | **N** | **%** | **N** | **%** | **N** | **%** | **N** | **%** | **N** | **%** |  |
| **EFP composite outcome** | **Total** | 32 | 100 | 16 | 100 | 16 | 100 | 15 | 100 | 7 | 100 | 8 | 100 | 17 | 100 | 9 | 100 | 8 | 100 |  |
|  | **Not achieved** | 1 | 3.1 | 0 | 0.0 | 1 | 6.3 | 1 | 6.7 | 0 | 0.0 | 1 | 12.5 | 0 | 0.0 | 0 | 0.0 | 0 | 0.0 |  |
|  | **All achieved** | 19 | 59.4 | 10 | 62.5 | 9 | 56.3 | 8 | 53.3 | 4 | 57.1 | 4 | 50.0 | 11 | 64.7 | 6 | 66.7 | 5 | 62.5 |  |
|  | **Not PPD** | 1 | 3.1 | 0 | 0.0 | 1 | 6.3 | 1 | 6.7 | 0 | 0.0 | 1 | 12.5 | 0 | 0.0 | 0 | 0.0 | 0 | 0.0 |  |
|  | **Not BoP** | 9 | 28.1 | 5 | 31.3 | 4 | 25.0 | 4 | 26.7 | 3 | 42.9 | 1 | 12.5 | 5 | 29.4 | 2 | 22.2 | 3 | 37.5 |  |
|  | **Not PPD, Bop/SUP** | 2 | 6.3 | 1 | 6.3 | 1 | 6.3 | 1 | 6.7 | 0 | 0.0 | 1 | 12.5 | 1 | 5.9 | 1 | 11.1 | 0 | 0.0 |  |
| **Derks et al. composite outcome** | **Total** | 32 | 100 | 16 | 100 | 16 | 100 | 15 | 100 | 7 | 100 | 8 | 100 | 17 | 100 | 9 | 100 | 8 | 100 |  |
|  | **Not achieved** | 1 | 3.1 | 0 | 0.0 | 1 | 6.3 | 1 | 6.7 | 0 | 0.0 | 1 | 0.0 | 0 | 0.0 | 0 | 0.0 | 0 | 0.0 |  |
|  | **All achieved** | 18 | 56.3 | 10 | 62.5 | 8 | 50.0 | 7 | 46.7 | 4 | 57.1 | 3 | 57.1 | 11 | 64.7 | 6 | 66.7 | 5 | 62.5 |  |
|  | **Not PPD** | 1 | 3.1 | 0 | 0.0 | 1 | 6.3 | 1 | 6.7 | 0 | 0.0 | 1 | 0.0 | 0 | 0.0 | 0 | 0.0 | 0 | 0.0 |  |
|  | **Not BoP/SUP** | 8 | 25.0 | 4 | 25.0 | 4 | 25.0 | 3 | 20.0 | 2 | 28.6 | 1 | 28.6 | 5 | 29.4 | 2 | 22.2 | 3 | 37.5 |  |
|  | **Not REC** | 1 | 3.1 | 0 | 0.0 | 1 | 6.3 | 1 | 6.7 | 0 | 0.0 | 1 | 0.0 | 0 | 0.0 | 0 | 0.0 | 0 | 0.0 |  |
|  | **Not BoP and REC** | 1 | 3.1 | 1 | 6.3 | 0 | 0.0 | 1 | 6.7 | 1 | 14.3 | 0 | 14.3 | 0 | 0.0 | 0 | 0.0 | 0 | 0.0 |  |
|  | **Not PPD, Bop/SUP** | 2 | 6.3 | 1 | 6.3 | 1 | 6.3 | 1 | 6.7 | 0 | 0.0 | 1 | 0.0 | 1 | 5.9 | 1 | 11.1 | 0 | 0.0 |  |

N – number; CAL – clinical attachment level; PPD – pocket probing depth; BoP - bleeding on probing; SUP – suppuration on probing; REC - mucosal recession buccal; % - percentage.
